# Community-based care for healthy ageing: lessons from Japan

**DOI:** 10.2471/BLT.18.223057

**Published:** 2019-06-03

**Authors:** Junko Saito, Maho Haseda, Airi Amemiya, Daisuke Takagi, Katsunori Kondo, Naoki Kondo

**Affiliations:** a Department of Health Education and Health Sociology, School of Public Health, The University of Tokyo, Room S310, Faculty of Medicine Building #3, Hongo 7-3-1, Bunkyo-ku, Tokyo, 113-0033, Japan.; b Center for Preventive Medical Sciences, Chiba University, Chiba, Japan.

## Abstract

**Problem:**

The measures for long-term care prevention that the Japanese government had introduced in 2006 were unsuccessful because of the failures to identify high-risk individuals and to enrol enough participants in the community prevention programme.

**Approach:**

The Japanese government shifted its primary strategy from a high-risk strategy to a community-based population strategy in 2015, by reforming the Long-term Care Insurance Act. This act is focusing on community-based care and social determinants of health. The Act and the government’s plans for long-term care prevention are inspired by a social participation intervention called *ikoino saron*, that is gathering salons for people older than 65 years. These salons, managed by local volunteers, are held once or twice a month in communal spaces within walking distance of community members’ homes and have a low participation fee. At the gatherings, older people can meet and interact with others through enjoyable, relaxing and sometimes educational programmes.

**Local setting:**

Japan has the world’s largest ageing population, with 27.7% (35.2 million/126.7 million) of people older than 65 years.

**Relevant changes:**

Studies have shown that participation in the salons was associated with a halved incidence in long-term care needs and about one-third reduction in the risk of dementia onset. Evidence also suggests that financially vulnerable older adults were more likely to participate in such interventions. In 2017, 86.5% (1506/1741) of the Japanese municipalities had implemented the salons.

**Lessons learnt:**

Integrated care for long-term care prevention should consider interventions targeting the whole community in addition to high-risk individuals.

## Résumé

**Problème:**

Les mesures pour la prévention des soins de longue durée qui avaient été mises en place par le gouvernement japonais en 2006 ont été inefficaces, en raison de l'impossibilité d'identifier les personnes à haut risque et de faire participer suffisamment de personnes au programme de prévention communautaire.

**Approche:**

Le gouvernement japonais a changé sa stratégie première, d'abord axée sur les personnes à haut risque, pour l'orienter en 2015 vers une prise en charge dans la communauté, en réformant la loi relative à l'assurance pour soins de longue durée. Cette loi se concentre sur la prise en charge dans la communauté et sur les déterminants sociaux de la santé. Elle s'inspire, comme les plans du gouvernement pour la prévention des soins de longue durée, d'une intervention pour la participation sociale appelée *ikoino saron*, qui consiste en des salons de réunion pour les personnes de plus de 65 ans. Ces salons sont organisés une ou deux fois par mois par des bénévoles locaux dans des espaces collectifs situés à une courte distance de marche, à un coût modique. Ils permettent aux personnes âgées de se réunir et d'interagir avec d'autres personnes autour de programmes de loisirs, de détente et parfois d'études.

**Environnement local:**

Le Japon est le pays du monde qui compte la population la plus âgée, avec 27,7% (35,2 millions/126,7 millions) de personnes de plus de 65 ans.

**Changements significatifs:**

Des études ont montré que la participation à ces salons était associée à une réduction de moitié de l'incidence des besoins en soins de longue durée et d'environ un tiers du risque de survenue de démence. Les données suggèrent également que les adultes âgés vulnérables sur le plan financier sont plus susceptibles de participer à ces interventions. En 2017, 86,5% (1506/1741) des municipalités japonaises avaient mis en place ce type de salons.

**Leçons tirées:**

La prise en charge globale pour la prévention des soins de longue durée devrait envisager des interventions axées sur l'ensemble de la communauté en plus des personnes à haut risque.

## Resumen

**Situación:**

Las medidas de prevención de cuidados a largo plazo que el gobierno japonés había introducido en 2006 no tuvieron éxito debido a que no se identificaron a las personas de alto riesgo ni se inscribió a un número suficiente de participantes en el programa de prevención comunitaria.

**Enfoque:**

El gobierno japonés cambió su estrategia primaria de una estrategia de alto riesgo a una estrategia de población basada en la comunidad en 2015, mediante la reforma de la Ley del Seguro de cuidado a largo plazo (Long-term Care Insurance Act). Esta ley se centra en la atención basada en la comunidad y en los determinantes sociales de la salud. La ley y los planes del gobierno para la prevención de los cuidados a largo plazo se inspiran en una intervención de participación social llamada *ikoino saron*, que reúne salones para personas mayores de 65 años. Estos salones, gestionados por voluntarios locales, se celebran una o dos veces al mes en espacios comunes a poca distancia y tienen una baja cuota de participación. En las reuniones, las personas mayores pueden conocer e interactuar con otros a través de programas divertidos, relajantes y a veces educativos.

**Marco regional:**

Japón tiene la mayor población de personas mayores del mundo, con un 27,7 % (35,2 millones/126,7 millones) de personas mayores de 65 años.

**Cambios importantes:**

Los estudios han demostrado que la participación en los salones se asoció con una incidencia reducida a la mitad en las necesidades de cuidados a largo plazo y una reducción de aproximadamente un tercio en el riesgo de aparición de la demencia. La evidencia también sugiere que los adultos mayores vulnerables económicamente tenían más probabilidades de participar en tales intervenciones. En 2017, el 86,5 % (1.506/1.741) de los municipios japoneses habían implementado los salones.

**Lecciones aprendidas:**

La atención integrada de prevención de cuidados a largo plazo debería considerar intervenciones dirigidas a toda la comunidad, además de a las personas de alto riesgo.

## ملخص

**المشكلة:**

لم تكلل تدابير الوقاية طويلة الأمد للرعاية التي قدمتها الحكومة اليابانية في عام 2006 بالنجاح، وذلك نتيجة الإخفاقات في تحديد الأفراد المعرضين للمخاطر العالية وتسجيل عدد كافٍ من المشاركين في برنامج الوقاية المجتمعي.

**الأسلوب:**

قامت الحكومة اليابانية بتبديل استراتيجيتها الأساسية من استراتيجية عالية الخطورة إلى استراتيجية سكانية قائمة على المجتمع في عام 2015، من خلال إصلاح قانون تأمين الرعاية طويلة الأمد. يركز هذا القانون على الرعاية المجتمعية والمحددات الاجتماعية للصحة. إن القانون وكذلك خطط الحكومة للوقاية طويلة الأمد للرعاية مستوحاة من تدخل للمشاركة الاجتماعية يُطلق عليه *ikoino saron *، وهو عبارة عن تجمع لصالونات من الأشخاص الذين تزيد أعمارهم عن 65 عاماً. تُدار هذه الصالونات بواسطة متطوعين محليين، وتُقام مرة أو مرتين في الشهر في مناطق شعبية مشتركة على مسافة قريبة للسير، وتتطلب رسوماً منخفضة للمشاركة. يمكن لكبار السن الالتقاء والتفاعل مع الآخرين في هذه التجمعات، من خلال برامج ممتعة ومريحة وقد تكون تعليمية أحياناً.

**المواقع المحلية:**

تمتلك اليابان أكبر تعداد من المسنين في العالم، حيث أن 27.7% من السكان (35.2 مليون/126.7مليون) تزيد أعمارهم عن 65 عاماً.

**التغيّرات ذات الصلة:**

أظهرت الدراسات أن المشاركة في الصالونات كانت مرتبطة بانخفاض احتياجات الرعاية طويلة الأمد إلى النصف، وانخفاض بمقدار الثلث تقريباً في خطر ظهور الخرف. تشير الدلائل أيضًا إلى أن البالغين كبار السن من ذوي الاحتياج المالي، مالياً كانوا أكثر احتمالية للمشاركة في مثل هذه التدخلات. في عام 2017، قامت 86.5% (1506/1741) من البلديات اليابانية بتنفيذ هذه الصالونات.

## 摘要

**问题:**

日本政府在 2006 年采取的长期护理预防措施因未能识别高危人群和招募足够的社区预防项目参与者而失败。

**方法:**

通过改革《长期护理保险法》(Long-term Care Insurance Act)，日本政府在 2015 年将其基本战略从高危群体战略转变为以社区为基础的人口战略。该法案强调基于社区的护理与健康的社会决定因素。该法案和政府的长期护理预防计划的灵感来自于一项名为 *ikoino saron* 的社会参与干预项目，该项目为 65 岁以上的人群举办沙龙。这些沙龙由当地志愿者管理，每月在公共场所举办一两次，步行可达，参与费用较低。在聚会上，老年人可以通过愉悦轻松的项目与他人会面和互动，这些项目时而会颇具教育意义。

**当地状况:**

日本是世界上老龄化人口最多的国家，65 岁以上人口占比 27.7%（3520 万/1.267 亿）。

**相关变化:**

研究表明，参加沙龙与长期护理需求的发病率降低一半、痴呆症发病风险减少约三分之一有关。证据仍表明，经济困难的老年人更有可能参与此类干预项目。2017 年，86.5％（1506/1741）的日本城市已开展沙龙。

**经验教训:**

长期护理预防的综合护理应考虑针对除高危群体以外的整个社区的干预措施。****

## Резюме

**Проблема:**

Меры по профилактике долгосрочного ухода, внедренные японским правительством в 2006 году, потерпели неудачу из-за того, что не удалось выявить лиц с высоким риском и включить достаточное количество участников в профилактическую программу по месту жительства.

**Подход:**

Правительство Японии сменило ранее существовавшую основную стратегию высокого риска на стратегию медицинского обслуживания населения по месту жительства в 2015 году, проведя реформу закона «О страховании долгосрочного ухода». Этот закон уделяет основное внимание медицинскому обслуживанию по месту жительства и социальным факторам здоровья. Сам закон и планы правительства по профилактике долгосрочного ухода были вдохновлены социальным времяпрепровождением, так называемым *икоино сарон*, то есть салонами для людей старше 65 лет. Такие салоны проводятся раз или два в месяц под руководством добровольцев в общественных помещениях в шаговой доступности за символическую плату. На этих собраниях пожилые люди могут встречаться и общаться в рамках развлекательных, досуговых и иногда обучающих программ.

**Местные условия:**

Япония характеризуется самым большим количеством стариков в мире, 27,7% (35,2 миллиона из 126,7) населения старше 65 лет.

**Осуществленные перемены:**

Исследования показали, что участие в таких салонах вдвое уменьшает потребность в долгосрочном уходе и снижает риск развития деменции почти на треть. Есть также подтверждения того, что финансово уязвимые пожилые люди более охотно участвуют в таких мероприятиях. В 2017 году салоны стали проводиться в 86,5% муниципалитетов Японии (в 1506 из 1741).

**Выводы:**

Комплексный уход как профилактика долгосрочного ухода должен включать мероприятия, направленные на все сообщество, а не только на лиц с высоким уровнем риска.****

## Introduction

Japan has the world’s largest ageing population. In 2017, 27.7% (35.2 million/126.7 million) of people living in Japan were older than 65 years. Over the years, the Japanese government has reformed its policies to respond to the need of the ageing population and to prevent long-term care. In 2006, the government implemented measures aimed to identify frail or semi-frail older adults (that is, 65 years or older) and provide early preventive care programmes for functional decline, to delay dependence on long-term care. The measures consisted of identifying older people with disability risks, by screening them, mainly at regular health check-ups, using a validated one-page questionnaire (Kihon checklist).^1^ Identified high-risk individuals were subsequently referred to free community prevention programmes.

However, the measures failed to identify high-risk individuals and participation in community programmes was low. Based on available evidence, the government estimated that approximately 5% of the total older population was at risk, and therefore should be the target of preventive care. However, in 2014, by the ninth year of strategy implementation, only 0.8% (267 654/32 824 841) of older adults had joined the community prevention programme.^2^ This result was due to the low participation in the screening process for functional difficulties: only 34.8% (11 408 862/32 824 841) of older people participated, a lower percentage than that for regular health check-ups (41.5% for 65–74-year-old people).^2^ Although supportive evidence is not available, we speculate that physical and environmental barriers and the lack of support to overcome these barriers, such as incentives and transportation, may explain the low participation. The low screening participation could also increase inequities in preventive service provision. A community-based survey identified that the proportion of socially disadvantaged people undergoing health check-ups was low.^3^ Moreover, the screening programme created ethical debates because the Japanese government categorized the older adults identified as frail as “special elderly” (*tokutei koureisha*). Some researchers and policy-makers were concerned about potential labelling and stigmatization, and in 2010, the government changed the name to “target individuals for secondary prevention programmes” (*niji-yobou taishousha*).

The low participation in the community prevention programmes resulted in limited attributable impact. In theory, even if the government succeeded in providing the programme to all eligible persons, these only represented 5% of the total older population. However, work on disease prevention, suggests that the distribution of disease and risk is generally a continuum, without an exact boundary between the normal and abnormal and that people developing a disease could be identified as normal in a screening programme.^4^ In Japan, half of those who developed functional decline did not belong to the high-risk or special elderly group before their functional decline started.^5^ The government recognized the issues associated to the secondary prevention measure, that is, difficulties in maintaining participants’ motivation and high discontinuance rates and hence revised its policies for preventing long-term care.^6^

Here we describe the country’s current strategy and we focus on a social participation intervention called *ikoino saron*, that is, salons where older people can gather.

## Current strategy

In response to the increasing awareness on health inequality, the second term of the National Health Promotion Movement: Health Japan 21 (2013–2022), started including the social determinants of health. Specifically, public long-term care prevention plans now focus on promoting social participation and preventing isolation of older people, since isolation has been identified as a strong risk factor for long-term care and premature mortality.^7^^,^^8^

In 2015, the government reformed the Long-term Care Insurance Act, by changing its primary strategy for long-term care prevention from a high-risk strategy to a community-based population strategy. The new strategy aims to build a community that can seamlessly provide preventive, medical and long-term care, welfare and housing services to all individuals. Based on the population strategy for long-term care prevention, central and local governments have promoted community activities, such as salons, to facilitate group participation and encourage social activities among older adults.

### The salons

The current Act and health promotion plan have been inspired by a project started in 2007 in the municipality of Taketoyo. The municipality, in collaboration with citizen volunteers and researchers, established social gathering opportunities for older adults. At these gatherings, older people can meet and interact with others through enjoyable, relaxing and sometimes educational social programmes, such as arts, crafts, music, health education seminar and physical and brain exercises.^9^ In 2013, there were 10 salons across Taketoyo and more than 10% (875/8062) of the eligible population attended these salons. Once a salon is established, local volunteers manage it with partial financial and administrative support from the townhall. The salons are held once or twice per month in communal spaces and a session last about two hours. Typically, 20 to 60 older adults attend one session, but large events may attract up to 100 people.^9^ To ensure accessibility and equal opportunities, the salons are within walking distance for most of the participants from their homes and the participation fee is only 100 yen (about 1 United States dollar) per visit. The project aims to provide a variety of activities that both promote health and enrich life, and to foster community-level social capital by encouraging community engagement.^9^

#### Relevant changes

In 2017, this salon-type community interventions were used in 86.5% (1506/1741) of the municipalities in Japan.^10^ Two studies in Taketoyo estimated that participation in the salon was associated with a halved incidence in long-term care needs and about one-third reduction in the risk of dementia onset.^11^^,^^12^ In a survey from the municipality of Tokai, 88 salon participants (out of 187 surveyed) answered that the salon increased their opportunities to go out, 117 (out of 188) responded an increased interaction with others and 56 (out of 183) responded that they were more likely to start participating in other social activities in the community.^13^ Furthermore, in seven municipalities, the proportion of high-risk individuals who participated in the salon to the total older population was almost twice as high as the proportion of high-risk individuals who participated in nationwide conventional secondary prevention programmes, based on the high-risk strategy (1.5%; 1535/100 593 versus 0.8%; 267 654/32 824 841).^14^ The difference could be due to the fact that the salons target all older people, including people who have limited access to adequate medical or social welfare services, as well as the low participation fee and the short distance from home to the venues. In 2007, the proportion of salon participants from low-income groups in Taketoyo was higher than that from high-income groups (8.0%; 6/75 versus 5.5%; 16/293 for men, and 19.0%; 47/247 versus 6.5%; 2/31 for women).^9^ These results suggest that salon-type community interventions may reduce the inequalities in social interactions.

## Lessons learnt

Shifting from a high-risk strategy to a population strategy involving multidisciplinary community collaborations has been successful in Japan. We learnt that for community-based integrated care systems to succeed, collaboration between community members and diverse service providers was indispensable. For instance, community members collaboration with local government staff was crucial for the sustainability of the interventions. The collaboration allowed community members to create or modify their own community welfare services in line with their needs and local situations (Box 1).

Box 1 **Summary of main lessons learnt**

• Integrated care for long-term care prevention should consider interventions targeting the community rather than only high-risk individuals.

• Salon-type community interventions proved effective in reducing long-term care needs and dementia, and may help reduce health inequalities.

• Multidisciplinary collaborations among diverse service providers and community members are indispensable for providing community-based care.

We also learnt that quantitative health equity assessments and visualizing the results in an easily understandable manner were useful in identifying and prioritizing problems, as well as sharing community goals of local actions and policies with service providers and community members. The Japan Gerontological Evaluation Study initiative have developed the Health Equity Assessment and Response Tool, in collaboration with the World Health Organization (WHO) Kobe Centre, which developed the urban version of the tool. The tool includes indicators for social determinants of health and allows users to assess health inequality within the city or across cities. This online tool has been used by local care providers to show trends in levels of long-term care risks and community resources for interventions.^15^

In 2017, WHO published the *Guidelines on integrated care for older people*, to provide guidance on preventing, slowing or reversing the decline of the intrinsic capabilities of older individuals and maximizing their functional abilities.^16^ The guidelines make evidence-based recommendations for the comprehensive assessment of the health status of older people and delivery of integrated health care. Most of the guidelines recommendations involve secondary prevention measures, that is, identifying frail people aged 60 years or older, and providing them with preventive care. However, as supported by Japan’s experience, secondary prevention measures or screening of high-risk individual needs effective screening measure to identify high-risk individuals, effective interventions to mitigate possible risks and effective means to deliver the intervention to high-risk individuals.^4^

We suggest, with the support of the empirical evidence gathered,^9^^, ^^11^^–^^15^ that integrated care for long-term care prevention should include more community-organized interventions for the whole community. To build local organizational networks for providing such care, health-care workers and organizations should be actively involved. The Japanese concept of community-based integrated care corresponds to local governance mechanisms in WHO’s ongoing programmes, including Healthy Cities and Healthy Ageing. The concept is also in line with the three recommendations of the final report of the WHO Commission on Social Determinants of Health, that is, improving daily living conditions, establishing good governance to secure equitable resource allocation and making health equity assessment. Eventually, the concept would help achieve universal health coverage. With these lessons from Japan, we suggest that WHO adds the perspectives of community-based care and social determinants of health to integrated care strategies.
